# Exercise-Induced Irisin, the Fat Browning Myokine, as a Potential Anticancer Agent

**DOI:** 10.1155/2019/6561726

**Published:** 2019-04-01

**Authors:** George-Emmanuel Maalouf, Diala El Khoury

**Affiliations:** Department of Sciences, Notre Dame University-Louaize, P.O. Box 72, Zouk Mikael, Lebanon

## Abstract

Irisin is a recently discovered myokine that plays an important role in fat metabolism through the browning of white adipose tissue. This myokine is usually secreted after exercise by improving energy balance and has shown great potential as a possible treatment for some metabolic diseases such as obesity, insulin resistance, and inflammation. Obesity has been linked to a higher incidence of some cancers. Furthermore, some studies have shown irisin to have direct positive effects on different types of cancers. Although it is hard to relay conclusions from *in vitro* to *in vivo* studies, the majority of the available data favor irisin as a potential substance for cancer regression through reducing proinflammatory markers linked to obesity. However, some controversies remain on the exact benefits of irisin on cancer with some studies showing no or even a negative effect of irisin on cancer. This review summarizes these 2 differing viewpoints and synthesizes them to form a clearer picture of exercise-induced irisin's effects on cancer.

## 1. Introduction

Obesity, along with the metabolic diseases it causes, has become an increasingly growing problem that has devastating economic, health, and societal effects. From insulin resistance and hyperlipidemia to cardiovascular diseases and cancer, obesity has been associated with many of the leading causes of death worldwide [1, 2]. Despite its prevalence, excess weight can be greatly reduced by following a healthy diet and a good exercise routine [1]. In fact, exercise is known to exert beneficial effects on all body systems. Studies have repeatedly proven the positive effects exercise has on the cardiovascular, respiratory, and skeletomuscular systems [3]. Exercise induces these changes by the release of hormones and myokines from the skeletomuscular system in the body [4]. Some of these myokines include BDNF, FGF-21, IL-15, and irisin [4, 5]. Indeed, one of these myokines, irisin, which was only recently discovered, has been shown to have numerous benefits not only in fighting metabolic diseases but also in combating cancer [4]. Recent reviews in the literature link irisin and exercise, concluding that irisin increases after exercise, however only transiently [6]; irisin, obesity, and metabolic diseases have also been linked [7], but no review summarizes the studies that show the effect of irisin on cancer or cancer biology. An investigation into the available literature shows that irisin has direct effects on different types of cancers. However, some controversies remain on the exact benefits of irisin on cancer with some studies showing no or even a negative effect of irisin on cancer. This review summarizes these 2 differing viewpoints and synthesizes them to form a clearer picture of irisin's effects on cancer and questions if irisin is the missing link between obesity, exercise, and cancer.

## 2. Methods

The articles listed in Section 3.4 were selected based on relevancy from PubMed and Google Scholar databases. Initial results yielded 56 articles on PubMed and 9280 articles on Google Scholar where “Irisin” and “Cancer” were used as keywords. After reading all the abstracts to refine the selection, only 17 articles were found to directly link irisin with cancer as these articles mentioned the direct effect of irisin on cancer cell lines *in vitro* and the effects of serum irisin in cancer patients *in vivo*.

## 3. Results

From the 17 articles selected and reviewed for the “irisin and cancer,”

- Thirteen articles (Table 1) favoured irisin as a myokine with a role in carcinogenesis or cancer therapeutics (7 studies were conducted *in vitro*, and 6 studies were performed *in vivo*)
- Four articles considered that irisin has no or adverse effect on cancer progression

### 3.1. Irisin

Irisin is released through the cleavage of FNDC5, a polypeptide protein containing 212 amino acids. This protein is cleaved at the *N*-terminal releasing irisin, originally named after the Greek goddess Iris, into the blood [21]. This cleavage is initiated by muscle contraction through an unknown proteasome [21]. Irisin levels are increased after acute exercise and bind to an unidentified receptor on the adipose tissue, which leads to significant weight loss and decrease in total body energy [21].

Recent studies of its function have determined that its beneficial effects derive from its ability of browning white adipose cells. The molecular cascade that ties irisin and the browning of white fat is the following: first, exercise increases the expression of PGC-1*α* or peroxisome proliferator-activated receptor (PPAR-*γ*) coactivator. This in turn increases the expression of FNDC5, which, as stated before, releases irisin. Irisin then increases the mRNA expression of UCP1, a transmembrane protein that decreases the proton gradient generated by oxidative phosphorylation [21].

### 3.2. Irisin and Exercise

Since irisin was linked with muscle contraction, studies were conducted to measure irisin levels after exercise. Several studies were conducted to measure the levels of irisin after exercise especially in overweight individuals. The selected articles of this section are based on recent studies conducted between 2014 and 2017 showing the relationship of irisin and exercise; the studies were performed on individuals of various body types and on mice. A murine study concluded that a significant increase in irisin levels and UCP1, which leads to increased thermogenesis in the white adipose tissue, was seen after rats were subjected to resistance exercise [22]. Another study conducted on humans indicated a significant increase in irisin levels among obese youth after exercise. However, this increase was observed directly after aerobic exercise while no change was observed after resistance exercise [23]. Other studies linked irisin levels with BMI, obesity, and leptin, where obese children who had undergone a physical activity program showed a significant increase in levels of irisin and leptin. This suggests that irisin could possibly link the skeletomuscular system with the adipose tissue [24]. A murine study conducted on high-fat diet-induced obese mice concluded that mice exposed to intravenous irisin gained similar benefits as those who had exercised. This study also showed that these two groups had improved insulin resistance and levels of reproductive hormones and improved ovarian follicle health [25]. Irisin was also found to induce muscular hypertrophy when injected into mice after activation of satellite cells and increase protein synthesis [26]. Lastly, in a study conducted on healthy human subjects, irisin and lactate levels were positively correlated and both increased with higher exercise workload, confirming the researchers' hypothesis that irisin is tied to muscle energy demands [27]. This correlation could suggest that the increased strain on the muscles and low ATP may signal irisin release. Interestingly, individuals who were able to reach a higher VO_2max_ and thus work at a higher exercise workload produced higher levels of irisin after exercise [27]. These studies show a clear pattern between irisin and exercise where exercise induced a significant increase in irisin secretion.

### 3.3. Irisin and Obesity

Irisin, originating from the white adipose tissue in mice, is thought to form around 30% of total body irisin, while in humans, muscle FNDC5/irisin expression is 100–200 times higher than in the white adipose tissue [28]. Another interspecies difference irisin shows is that its browning effects in humans may be different than those in rodents. Indeed, irisin decreases browning-related genes in human preadipocytes but increases said genes in mature human adipocytes [6, 21].

Since irisin increases energy expenditure through its aforementioned effects on UCP1, it is expected that it should reduce body weight. Hence, it is paradoxical that obese individuals show increased irisin levels as compared to normal weight individuals. In fact, anorexic patients show as much as 30% reduced irisin levels compared to morbidly obese individuals. It is surmised then that while increased fat deposits increase the production of irisin, irisin itself can no longer exert its effects in a meaningful manner; thus, obese individuals may have irisin resistance, a condition not too different from leptin resistance, where increased leptin levels fail to enact their beneficial effects. In turn, leptin is also positively correlated with obesity and irisin itself [29]. Many studies revealed a link between irisin and obesity due to its secretion by the adipose tissue and have found a positive correlation between BMI and irisin [7].

### 3.4. Irisin and Cancer

**Figure 1:**
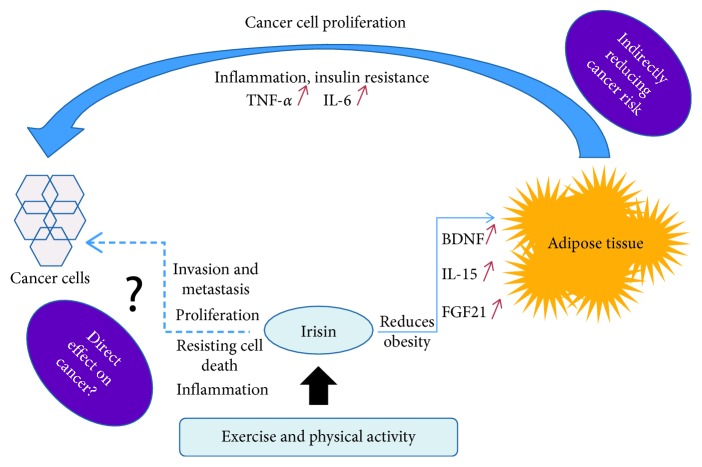
Diagram representing the putative relationship between exercise-induced release of irisin, obesity, and cancer. A possible anti-inflammatory role of irisin could also be inferred by its ability of browning fat cells, reducing obesity and thus reducing the inflammatory microenvironment. Irisin may also have a direct effect on other cancer hallmarks.

A large body of evidence has linked obesity to cancer because obesity leads to an increase in inflammatory markers (IL-6 and TNF‐*α*) [30], insulin resistance [31], and adipokine secretions [32], all of which favor tumor survival and proliferation [33], while exercise has also shown to have potential anti-inflammatory effects by reducing of TNF‐*α* expression [34]. Thus, since irisin is linked to obesity, it follows to hypothesize that irisin could be associated with cancer as well (Figure 1). Tying together obesity, irisin, and cancer, exercise, which helps combat obesity, also increases irisin levels [23]. Exercise‐induced irisin could be used as a determinant of the metabolic response to exercise in obese individuals to track any decrease in cancer risk linked to obesity [35]. To study irisin's effects on cancer, a study was conducted on human nonmalignant breast epithelial cells (MCF-10a), malignant breast epithelial cells (MCF-7), and malignant aggressive breast epithelial cells (MDA-MB-231). Upon exposure to irisin, the malignant breast tumor cell number significantly decreased as a result of increased caspase-3/7 activity and suppression of NF-κB activity. This shows that irisin could possibly reverse the cancer hallmark of resisting cell death [36, 37] by promoting caspase 3 activity and thus apoptosis. A significant decrease in cell migration compared to the control was also noted. Moreover, the malignant breast cells were exposed to doxorubicin, a chemotherapy agent, and irisin. When exposed to irisin, these cells showed a significant increase in doxorubicin sensitivity and a significant decrease in malignant cell viability and number. In fact, this increased doxorubicin sensitivity meant that less doses of doxorubicin were even more effective at producing the desired chemotherapy effects. Thus, irisin could play an important role in cancer therapy [10]. Another study on irisin and breast cancer showed that there was a negative correlation between serum levels of irisin and spinal metastasis of breast cancer. Irisin was found to have a protective effect on the bone, and these favorable bone qualities protected from metastasis of breast cancer [19]. This shows that irisin's effects also could reduce the metastatic and invasive hallmarks of cancer [36].

Yet another study suggested that increased levels of serum irisin reduced the risk of breast cancer development by 90%, and patients that had developed breast cancer had a significantly lower irisin serum levels than healthy individuals [9].

Aiming to uncover irisin's relation to other types of cancers, two studies conducted on lung cancer cells concluded that an increase in irisin levels led to a decrease in lung cancer cell proliferation, viability, and invasiveness by affecting the epithelial-mesenchymal transition (EMT), significantly decreasing the EMT markers *N*-cadherin and vimentin and increasing the expression of *E*-cadherin. This inhibition in EMT was related to the inhibition of the Snail pathway which is mediated by the PI3K/Akt pathway [8]. Irisin's effect on the PI3K pathway may also suggest an inhibitive role and a reason for the reduction in cancer cell proliferation. The second study conducted on osteocarcinoma cells also confirmed these findings and deduced that irisin was able to reverse IL-6-induced EMT by inhibition of the STAT3 pathway [18]. Another recent study conducted on pancreatic cancer cell lines also showed irisin's ability to inhibit EMT, viability, and proliferation of these cells by activation of the AMPK pathway. Irisin inhibits pancreatic cancer cell growth via the AMPK-mTOR pathway [20]. This ability of irisin to target the AMPK pathway may also suggest its role in reducing proliferation and altering cancer energy metabolism [37, 38]. A study conducted on prostate cancer cells subjected to different concentrations of irisin showed reduced prostate cancer cell viability [13]. Furthermore, a study on renal cell cancer suggested that irisin can be used as a biomarker for renal cancer diagnosis as irisin levels were significantly increased in patients with renal tumors; irisin too had higher specificity and sensitivity than other investigated biomarkers [11]. In a recent study conducted, patients with colorectal cancer showed low serum irisin levels compared to healthy individuals while individuals with high levels of irisin showed a 78% reduced risk of developing colorectal cancer (CRC). These findings could show that irisin could have protective qualities against development of CRC [14].

### 3.5. Controversies

Despite what appears to be a positive correlation between irisin and cancer, two studies conducted on hepatocellular carcinomas have suggested that irisin stimulated the proliferation and invasion of hepatocellular carcinoma tumors via activation of the PI3K/AKT pathway. This study also showed a reduction in doxorubicin cytotoxicity in the presence of irisin [16].The second study showed a significant increase in expression of hepatic irisin mRNA in individuals with hepatic carcinoma [12].

Furthermore, a murine study conducted to assess the relationship between cachectic factors and irisin in gastric cancer showed no significant difference in the expression of FNDC5 in gastric cancer tissues. However, FNDC5 expression was increased in the subcutaneous adipose tissue, and an overall increase in irisin serum levels was noted. Nevertheless, these increased serum irisin levels seen in this study failed to increase UCP1 expression in the white fat tissue, while increased irisin levels due to exercise do increase UCP1 expression [17]. Therefore, this shows that the increased irisin levels due to exercise operate differently than those seen in gastric cancer and possibly other types of cancer.

Moreover, a study conducted on several cancer cell lines where adhesion activity and colony numbers were measured failed to show any effects of irisin on the proliferation and malignant potential of these cell lines [15]. Indeed, these studies showed that perhaps the beneficial effects of irisin are cell- or tissue-specific and may not be observable in all cancer types.

## 4. Discussion

Exercise has shown its positive influence in numerous chronic diseases, especially obesity, but the direct path that causes these positive changes remained elusive [39]. Exercise has also shown its effect on several hallmarks of cancer [38]. No study has yet revealed the exact type and duration of exercise that should be performed to decrease cancer risk. In our present review, we shed the light on irisin as an exercise-secreted myokine, and we summarize the studies that show the effect of irisin on some of the hallmarks of cancer. Indeed, the studies conducted showed that cancer cells exposed to irisin presented an increase caspase activity, a suppression of NF-κB activity, thus a reduction of the “resisting cell death” hallmark [36]. Other studies showed a role of irisin in suppressing other hallmarks of cancer such as “sustaining proliferative signaling” [36] by targeting the PI3K/Akt pathway [16] or “evading growth suppressors” [36] via targeting the AMPK-mTOR pathway [20] or “activating invasion and metastasis” [36] by decreasing cell migration and inhibiting the epithelial-mesenchymal transition [18]. Irisin belongs indeed to the emerging group of myokines that are hypothesized to reduce cancer risk by lowering the basal systemic levels of cancer risk factors such as proinflammatory cytokines and adipokines [37, 40].

## 5. Conclusion

Further mechanistic studies are necessary to determine how irisin induced fat browning and obesity reduction may reduce carcinogenesis or cancer risk. As for the potential role of irisin in cancer therapeutics, more studies should be performed in order to determine the mode of administration of irisin for each cancer type. Nevertheless, obesity has become a worldwide epidemic disease, and many diseases related to obesity also present a steady rising, including insulin resistance, metabolic syndrome, type II diabetes, hypertension, chronic kidney disease, cardiovascular disease, heart failure, and cancer. Therefore, exercise-induced irisin deserves a closer inspection to further understand its direct role in reducing obesity and to elucidate its part in cancer prevention and therapeutics.

## Figures and Tables

**Table 1:** Studies that show the promising effects of irisin on different types of cancer.

Subject/animal/culture	Tumor/cell model	Treatment/groups	Main results	Authors' main conclusions	Reference
Lung cancer cells	A549 and NCI-H446 lung cancer cells	Treated with 0, 10, 20, or 50 nM irisin, respectively, for different time periods (0, 24, and 48 h)	(i) Over a range of concentrations (20–50 nM), significantly irisin inhibits A549 cells proliferation as detected by MTT assay (ii) Irisin may alter the expression of EMT markers in a concentration-dependent manner and indicate that the inhibitory effect of irisin on lung cancer cells invasion and migration may be associated with EMT.	(i) Inhibition of proliferation, migration, and invasion of osteosarcoma cells (ii) Suppression of IL-6-induced EMT in osteosarcoma cells (ii) targeting the STAT3/Snail signaling pathway	Shao et al. [8]

Female patients with invasive ductal breast cancer and healthy women	—	Two groups: 101 females with invasive ductal breast cancer and 51 healthy females	(i) Irisin discriminates between patients and healthy individuals at an optimal value of 3.21 *μ*g/ml (ii) Irisin levels were positively associated with tumor stage (iii) No significant association between irisin and tumor grade, and irisin and CEA, and CA15-3 and Her2/neu	(i) Irisin may serve as a novel biomarker for breast cancer diagnosis (ii) An understanding of irisin's role in health, and disease is lacking	Provatopoulou et al. [9]

Breast cancer cells	MDA-MB-231 cells and MCF-10a cells	MDA-MB-231 and MCF-10a cells were treated with human recombinant nonmodified irisin (INM) or human recombinant modified and active (glycosylated) irisin (IM)	(i) INM did not affect nonmalignant MCF-10a cell viability, but IM decreased it (ii) Malignant MDA-MB-231 cell viability was significantly reduced by INM, but not IM (iii) INM decreased cell number while IM did not (iv) Caspase-3/7 activity was significantly elevated when cells were treated with INM but not when treated with IM (v) IM enhanced Dox killing at 1.0 *μ*M, while INM enhanced it at all tested concentrations	(i) Irisin is a potential therapeutic agent for cancer (ii) Irisin may have an anti-inflammatory effect, counteracting the effects of TNF-*α* (iii) Irisin affects malignant cells without affecting nonmalignant cells	Gannon et al. [10]

Patients with renal cell cancer and healthy subjects	—	Two groups: 23 patients with renal tumor and 25 healthy individuals	(i) Significantly elevated FNDC5/irisin levels and CEA in patients with renal tumor (ii) FNDC5 levels showed higher sensitivity and specificity indexes when compared to CEA	(i) Irisin may be a useful marker in the diagnosis of cancer	Altay et al. [11]
Patients undergoing liver transplantation and deceased donors	—	Two groups: 18 patients with HCV-related HCC undergoing liver transplantation and 18 deceased liver donors	(i) Irisin mRNA expression was significantly higher in the liver of HCC patients than in liver donors (ii) SCD-1, NOTCH1, IL-6, and TNF-*α* were significantly higher in HCC patients than in donors (iii) Irisin mRNA correlated with the plasma lipid profile (triglycerides), DNL index, and PUFA/SFA ratios	(i) Irisin levels increase in hepatocellular carcinoma as a compensatory mechanism to limit cancer-induced lipogenesis. (ii) No correlation between hepatic irisin levels and plasma irisin levels, possibly due to plasma irisin levels depending on the sum total of the adipose tissue and skeletal muscle	Gaggini et al. [12]

Prostate cancer cells	DU-145 and PC3	Treated with 0.1, 1, 10, and 100 nM irisin	(i) Irisin reduced proliferation and cell viability of the DU-145 and PC3 cells when treated with 10 and 100 nM of irisin, respectively	Irisin has a cytotoxic effect on prostate cancer cells on both ± androgen receptors in a dose-dependent manner	Tekin et al. [13]

Patients with colon and rectal cancer	—	76 CRC patients and 40 healthy controls	(i) Patients with CRC have significantly reduced levels of irisin (ii) High serum irisin levels had a 78% reduced risk of developing CRC.	(i) Irisin could act as a potential serum diagnostic biomarker for CRC (ii) Irisin could be a protective factor in CRC development	Zhu et al. [14]

Endometrial, colon, thyroid, and esophageal cell lines	KLE and RL95-2, HT29 and MCA38, SW579 and BHP7, and OE13 and OE33	Cells were treated with irisin for a period between 24 and 36 hours	(i) No change in cell adhesion or colony number (ii) No effect on cell proliferation	(i) Irisin did not have an effect of cell proliferation or malignant potential of human and mouse obesity-related cancer cell lines	Moon and Mantzoros [15]

20 patients with hepatocellular carcinoma and hepatocellular carcinoma cells.	HepG2 and SMCC7721	Cells were treated with glycosylated and nonmodified irisin for 24 h.	(i) HepG2 and SMCC7721 viability and proliferation increased (ii) Doxorubicin cytotoxicity was reduced	(i) Irisin regulates liver cancer cell proliferation (ii) Irisin significantly increases the activation of the PI3K/AKT pathway (iii) Irisin reduces doxorubicin sensitivity	Shi et al. [16]

60 BALB/c mice	—	12 mice as controls and 48 mice receiving carcinogenic MNU	(i) No FNDC5/irisin expression was detected in cancerous stomach tissue (ii) Significant increase in FNDC5/irisin expression in subcutaneous adipose tissue after development of cancer	(i) Gastric tumors stimulated the release of FNDC5 leading to weight loss	Altay et al. [17]
Osteosarcoma cells	U2OS and MG-63 osteosarcoma cells	Osteosarcoma cells were treated with different doses of irisin (0, 25, 50, 100, and 200 ng/ml) for different times (12, 24, and 48 h) and were also treated with and without IL-6	(i) Irisin significantly suppressed osteosarcoma cell viability after 24 h (ii) Irisin significantly inhibited osteosarcoma cell proliferation after 48 h (iii) Irisin reversed the effect of IL-6 and suppressed EMT transition (iv) Irisin downregulated STAT3 phosphorylation (v) Irisin inhibits Snail expression via STAT3 pathway	(i) Irisin suppressed metastasis by the inhibition of EMT via the STAT3/Snail pathway (ii) Irisin suppressed the migration and invasion of osteosarcoma cells (iii) Irisin reversed the EMT induced by IL-6	Kong et.al. [18]

148 female patients with breast cancer	—	—	(i) Patients with spinal metastasis had significantly lower levels of serum irisin (ii) High serum irisin levels reduced the risk of spinal metastasis by 20%	(i) Irisin has protective qualities against the development of spinal metastasis (ii) Irisin can be used as a predictive marker for bone metastasis	Zhang et al. [19]

Pancreatic cancer cell lines	MIA PaCa-2 and Panc03.27	Cells were treated with different concentrations (0, 10, and 100 nM) of E-irisin and P-irisin for 2 weeks	(i) Reduced PaCa-2 and Panc03.27 viability (ii) Reduction in mobility and invasiveness (iii) Upgregulation of E-cadherin expression (iv) Increase in phosphorylation of AMPK	(i) Irisin suppressed invasion and migration of pancreatic cancer cells by inhibiting EMT	Liu et al. [20]
